# Inhibition of IAP’s and activation of p53 leads to caspase-dependent apoptosis in gastric cancer cells treated with Scutellarein

**DOI:** 10.18632/oncotarget.23202

**Published:** 2017-12-11

**Authors:** Venu Venkatarame Gowda Saralamma, Ho Jeong Lee, Suchismita Raha, Won Sup Lee, Eun Hee Kim, Sang Joon Lee, Jeong Doo Heo, Chungkil Won, Chang Keun Kang, Gon Sup Kim

**Affiliations:** ^1^ Research Institute of Life Science and College of Veterinary Medicine, Gyeongsang National University, Gazwa, Jinju, Republic Korea; ^2^ Department of Internal Medicine, Institute of Health Sciences, Gyeongsang National University School of Medicine, Jinju, South Korea; ^3^ Department of Nursing Science, International University of Korea, Jinju, Republic of Korea; ^4^ Gyeongnam Biological Resource Research Center, Korea Institute of Toxicology, Jinju, Gyeongsangnam 666-844, Republic of Korea; ^5^ Institute of Animal Medicine, College of Veterinary Medicine, Gyeongsang National University, Jinju, Republic of Korea

**Keywords:** apoptosis, gastric cancer, scutellarein, p53, IAPs

## Abstract

Gastric cancer is the fifth most common cancer and the third leading cause of cancer deaths worldwide. South Korea is in first place with 9,180 death alone attributed to gastric cancer in 2013. Plenty of literature suggests the evasion of apoptosis is implicated in neurodegeneration, autoimmune diseases, and tumors development due to dysregulation in the apoptotic mechanism. Reduced apoptosis or its resistance in cancer cells plays a significant role in carcinogenesis. It’s imperative to understand apoptosis, which provides the basis for novel targeted therapies that can induce cancer cell death or sensitize them to cytotoxic agents by regulating key factors like IAPs, MDM2, p53, caspases and much more. Studies have demonstrated that Scutellarein have the ability to inhibit several cancer cells by inducing apoptosis with both: Scutellarein monomers as well as scutellarein containing flavonoids. MTT results revealed that scutellarein inhibited cell viability in both dose and time dependent manner. Flow cytometry and western blot analysis showed that scutellarein induces apoptosis in both AGS and SNU-484 human gastric cancer cells and G2/M phase cell cycle arrest in SNU-484 cells. This study demonstrated that the Scutellarein on AGS and SNU-484 cells significantly inhibits cell proliferation and induces apoptotic cell death via down regulating MDM2 and activated the tumor suppresser protein p53, subsequently down regulating the IAP family proteins (cIAP1, cIAP2, and XIAP) leading to caspase-dependent apoptosis in AGS and SNU-484 cells.

## INTRODUCTION

Apoptosis has been widely appreciated and intensively studied in the past two decades as a major mechanism of regulated cell death. This form of regulated cell death, occurs during development and morphogenesis of eukaryotic cells and during pathological conditions, which eliminates damaged or non-essential cells without causing local inflammation from cell leakage. Apoptosis is a decisive mechanism complementary to proliferation, which maintenance tissue homeostasis by selective elimination of damaged or unwanted cells [1]. Apoptotic cells exhibit credible morphological changes including, nuclear condensation, DNA fragmentation, cell shrinkage as well as plasma membrane bubbling [2]. Apoptosis transpire mainly through two pathways. One of which is the extrinsic or death receptor pathway, which is triggered through the Fas death receptor, a member of the tumor necrosis factor (TNF) receptor superfamily.Also intrinsic or mitochondrial pathway, which occurs due to internal stimuli such as irreparable genetic damage, hypoxia, extremely high concentrations of cytosolic Ca^2+^ within the cell leads to the release of cytochrome-c from the mitochondria. Both the pathways converge at a common point where activation of effector caspases leads to cleavage structural molecules of cell [3]. There is much literature that suggests the evasion of apoptosis is implicated in neurodegeneration, autoimmune diseases, and tumors development due to dysregulation in the apoptotic mechanism. Reduced apoptosis or its resistance in cancer cells plays a significant role in carcinogenesis. The mechanisms by which obliqueness of apoptosis occurs in cancer cells mainly of three types: 1) Impaired death receptor signaling, 2) Disrupted balance of pro-apoptotic and anti-apoptotic proteins and 3) Reduced caspase activation [4]. Impairment of receptor function or down regulation of the receptor, as well as a reduced level in the death signals together contribute to impaired signaling and leads to reduction of apoptosis in cancer cell. The ratio of pro- and anti-apoptotic protein balance for apoptosis in cancer cells have been imbalanced due to over-or under-expression of a certain gene which dysregulated them to contribute to carcinogenesis by reducing apoptosis in cancer. The p53 protein, also called tumour protein 53 is one such gene which is encoded by tumour suppressor gene TP53 located at the short arm of chromosome 17 (17p13.1) [5, 6]. The p53 protein expression was down regulated in most of the cancer cells due to mutation or gain of oncogenic function which is not only involved in the induction of apoptosis but also playing critical role in cell cycle regulation, chromosomal segregation, DNA recombination and cellular senescence [4, 7, 8]. The inhibitor of apoptosis proteins (IAPs), a group of functionally and structurally similar proteins which are endogenous inhibitors of caspases [9]. IAPs can inhibit caspase activity by binding their conserved BIR domains to the active sites of caspases, by keeping the caspases away from their substrates or by promoting degradation of active caspases. Up regulation of several IAPs like NAIP (BIRC1), X-linked IAP (XIAP, BIRC4), Survivin (BIRC5), c-IAP1 (BIRC2), c-IAP2 (BIRC3), Apollon (BRUCE, BIRC6), Livin/MLIAP (BIRC7) and IAP-like protein 2 (BIRC8) have been reported in several cancer cells as well as in serum from cancer patients [10, 11]. Caspase mediated proteolysis is a critical element of the apoptotic process, which has been blocked in most of the cancer cells due to several regulatory factor’s like higher expression of IAPs, down regulation of p53 and many more. It’s imperative to understand apoptosis, which provides the basis for novel targeted therapies that can induce cancer cell death or sensitize them to cytotoxic agents by regulating these key factors [11–14]. The novel agents include those targeting the intrinsic Bcl-2 family pathway such as antisense bcl-2 oligonucleotides and the extrinsic pathway such as TNFR1 (Tumor necrosis factor receptor 1).There are several potent endogenous sets of genes and proteins that inhibit apoptosis. Targeting some of these dysregulated factors in cancer cells death as apoptosis-like the p53, IAPs, Bcl-2 family of proteins, and caspases activation will be decisive in cancer treatment [15].

Gastric cancer is the fifth most common cancer and the third leading cause of cancer deaths worldwide and South Korea is being in the first place by causing 9,180 gastric cancer death in 2013 [16–18]. However, due to late diagnosis and its lack of appropriate treatment, only palliative therapy has been recommended. Furthermore, chemotherapy has begun to prove its efficacy for adjuvant, pre-operative and postoperative therapies in past decades.Unfortunatly, its eminent limitations of having indefinite benefits and high toxicity still remain a problem [17]. Thus, there is an urgency to find an alternative strategy which causes cytotoxicity in cancer cells by causing a programed cell death and non-toxic effect to normal cells. Natural products are known to have potential anticancer effects by targeting the multiple cellular signaling pathways including apoptosis with minimal cytotoxicity towards normal cells [19–22].

Flavonoids are ubiquitous in nature, extensively present in vegetables and fruits, providing an essential link between diet and prevention of chronic diseases including cancer [23]. Scutellarein (5, 6, 7, 4’-tetrahydroxy flavone), a flavone glycoside belongs to the flavonoids family and it’s a hydrolyzed product of Scutellarin found in perennial herbs like *Scutellaria baicalensis*, *Scutellaria lateriflora*, *Scutellaria barbata.* The previous study’s demonstrated Scutellarein containing flavonoid extracts as well as monomer have to cover a broad spectrum of biological activities like antioxidant, anti-inflammatory and anticancer by inducing apoptosis [24–27]. In the present study, we scrutinize the potential of Scutellarein to attenuate the gastric cancer cell viability and its underlying molecular mechanism and its anticancer effect. To the best of our knowledge, the present study is the first report that elucidates the molecular mechanism of Scutellarein in inhibition cell growth and inducing apoptosis in human gastric cancer cells.

## RESULTS

### Scutellarein inhibited cell proliferation in AGS and SNU-484 gastric cancer cells

**Figure 1:**
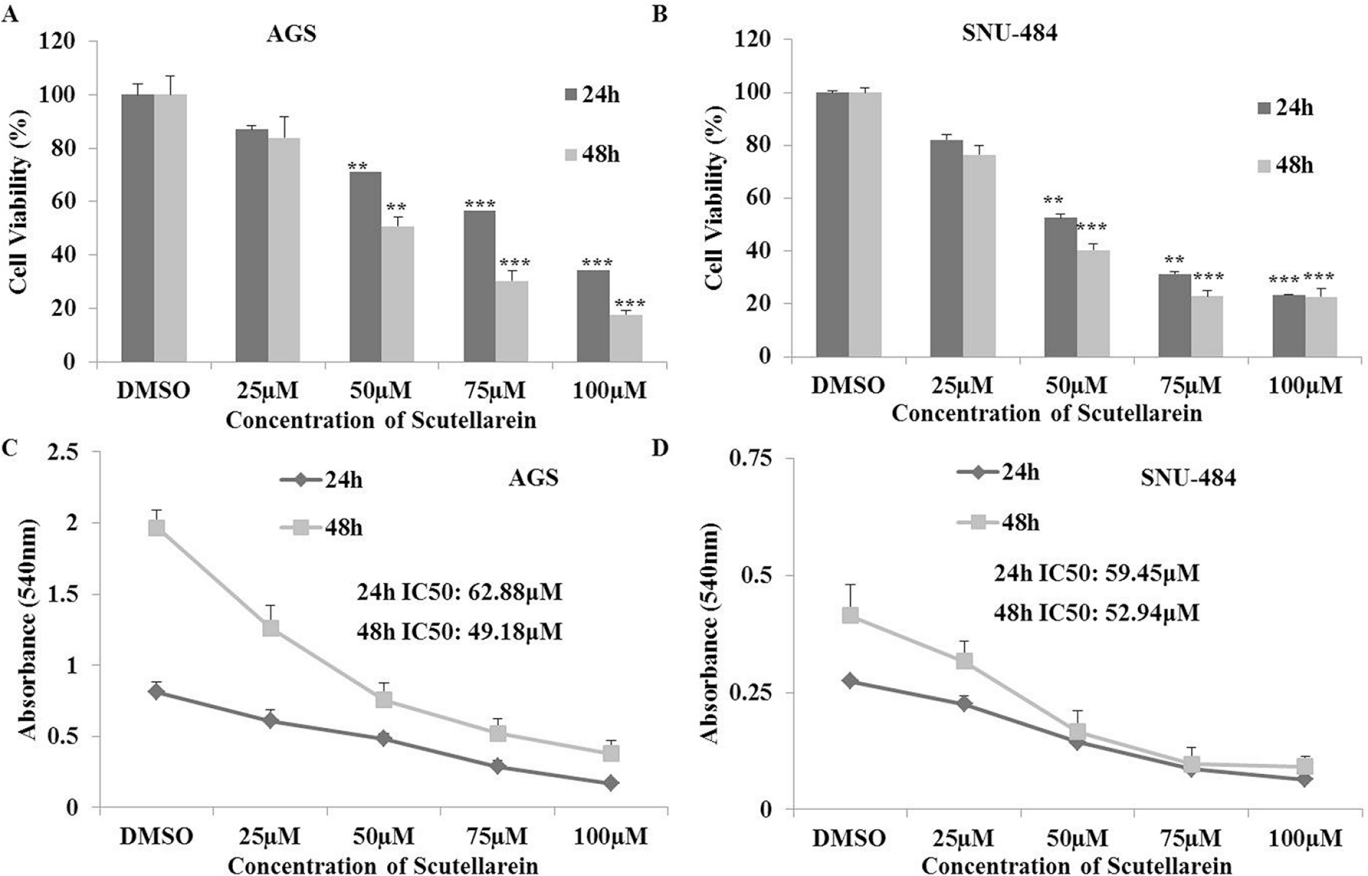
Inhibitory effects of Scutellarein on AGS and SNU-484 Gastric cancer cells (**A**–**B**) Both the gastric cancer cells were treated with indicated concentrations (0, 25, 50, 75 and 100 μM) of scutellarein for 24 h and 48 h. Cell viability was determined by a MTT assay. (**C**–**D**) Cell proliferation curves of AGS and SNU-484 cells treated scutellarein for 24 h and 48 h with or without Scutellarein. The data are expressed as the mean ± standard deviation (SD) of at least three independent experiments. (^**^P < 0.05, ^***^P < 0.01 compared to control).

MTT assay was carried out to quantify the inhibitory effect of scutellarein on AGS and SNU-484 gastric cancer cells. As shown in (Figure 1A–1D), scutellarein inhibited the proliferation of AGS and SNU-484 cells in a time and dose-dependent manner. It was noticed that the cell viabilities of each cell line at 24 h and 48 h reflected minute differences, implying that the cells respond to scutellarein within 24 h. Interestingly, at the highest dose of scutellarein (100 μM), cell viability of SNU-484 cells appeared to be independent of time (i.e. the drug effects are similar for each of the three indicated time points) but it was decreasing in AGS cell. Half-maximal inhibitory concentration (IC50) values are commonly used to evaluate the potency of a compounds, in which the lower the IC50 value, the more potent the compound is. The obtained results revealed that the IC50 values for AGS cells were 62.88 and 49.18 μM at 24 h and 48 h respectively, whereas the IC50 value of SNU-484 cells were 59.45 and 52.91 μM at 24 h and 48 h respectively. The inhibitory effect of Scutellarein is cancer specific because it did not demonstrate any cytotoxicity in normal cells [25, 27]. We chose three different concentrations (25, 50 and 100μM) whereas 25 μM being lowest inhibition concentration and 100 μM being highest inhibition concentration for further experiments.

### Scutellarein induced G2/M phase cell cycle arrest in SNU-484 cells but not in AGS cells

**Figure 2:**
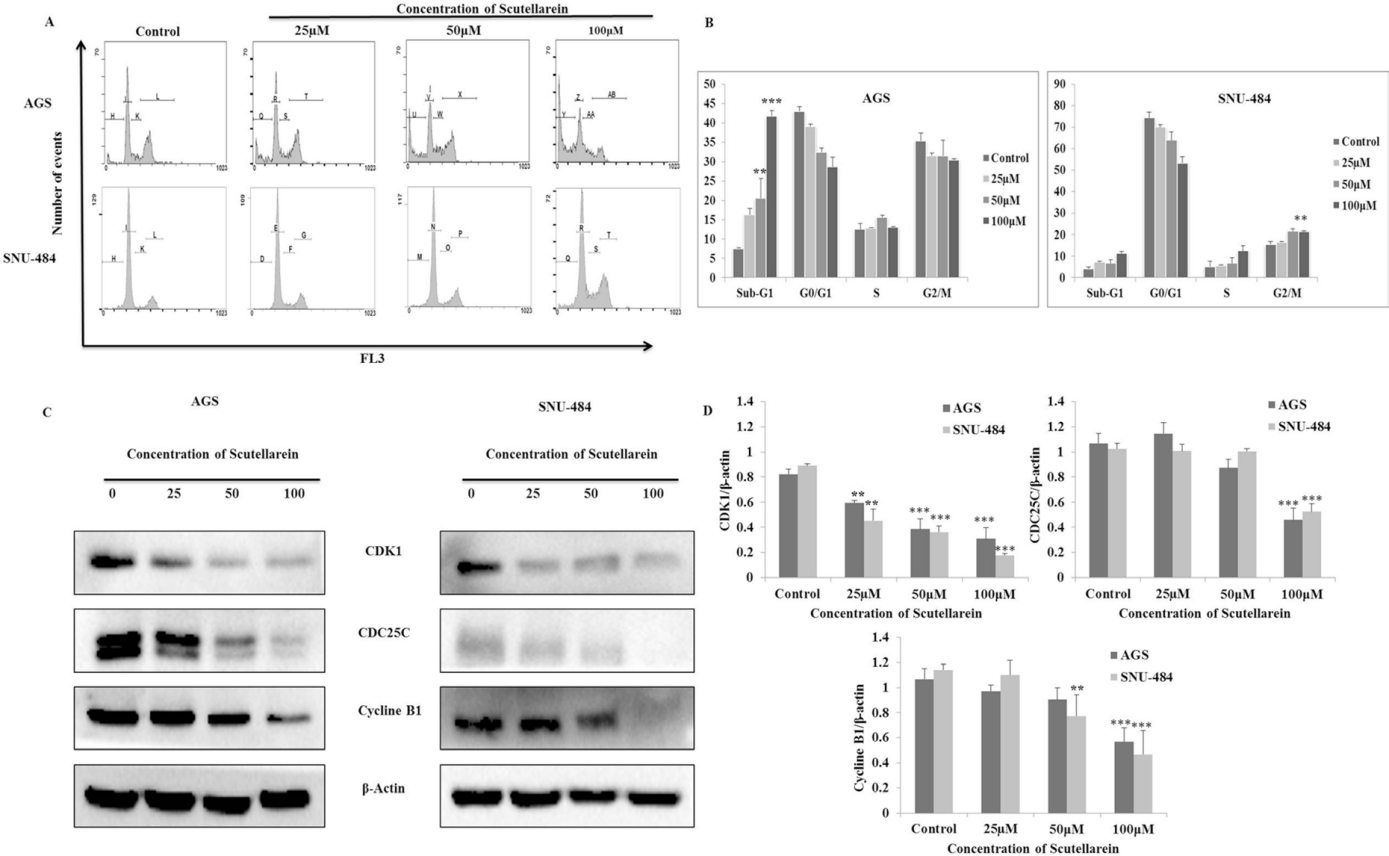
Regulatory effect of Scutellarein on cell cycle progression of AGS and SNU484 cells AGS and SNU-484cells were treated with indicated concentrations of scutellarein for 24 h. (**A**–**B**) Cell cycle distribution was determined by using Cytomics FC 500 (Beckman Coulter, Brea, CA, USA).The data were analyzed using CXP Software. (**C–D**) Effect of Scutellarein on cell cycle-related proteins (cyclin B1, CDK1 and CDC25C) expression level in A549 cells. Cells were treated with Scutellarein (0, 25, 50 and 100 μM) for 24 h. Cell lysates were subjected to SDS–PAGE and analyzed by Western blotting. Representative blots are shown. Densitometric analyses of the effect of Scutellarein on expression of cell cycle-related proteins level were represented. The data are expressed as the mean ± standard deviation (SD) of at least three independent experiments. (^∗^*P* < 0.05 ^∗∗∗^*P* < 0.01 compared to control).

Considering the fact that scutellarein inhibited cell proliferation, flow cytometric analysis on cell cycle progression was performed to determine the mechanism for anti-proliferative effect of scutellarein on the gastric cancer cells. Both AGS and SNU-484 cells were treated with three different concentrations of scutellarein (25, 50 and 100 μM) for 24 h. The distribution of cell cycle was analyzed using PI staining. As shown in Figure 2A and 2B, there was a significant amount of G2/M phase of cell accumulation in SNU-484 cells treated with 100 μM and slight increase in sub-G1 phase of cell population. Whereas in AGS cells treated with scutellarein, there was no cell cycle arrest in G2/M phase of cell cycle instead accumulation of dose dependent Sub-G1 phase of cell population indicating apoptotic cell death in AGS cells. Western blot result revealed that the expression of CDK1,CDC25C and cyclin B1 protein expression levels decreased in a dose-dependent manner, with significant inhibition occurring at 50 and 100 μM concentrations as shown in Figure 2C and 2D. In both cells the cell cycle regulation caused by scutellarein revealed significant decrease in G2/M phase cell cycle arrest proteins (*P* < 0.05). Taken together, scutellarein caused growth arrest in G2/M phase of cell cycle in SNU-484 cells, but not in AGS cells, signifying the differential regulation of scutellarein on cell cycle progression between the two cell lines.

### Scutellarein induces dose-dependent apoptosis in both AGS and SNU-484

**Figure 3:**
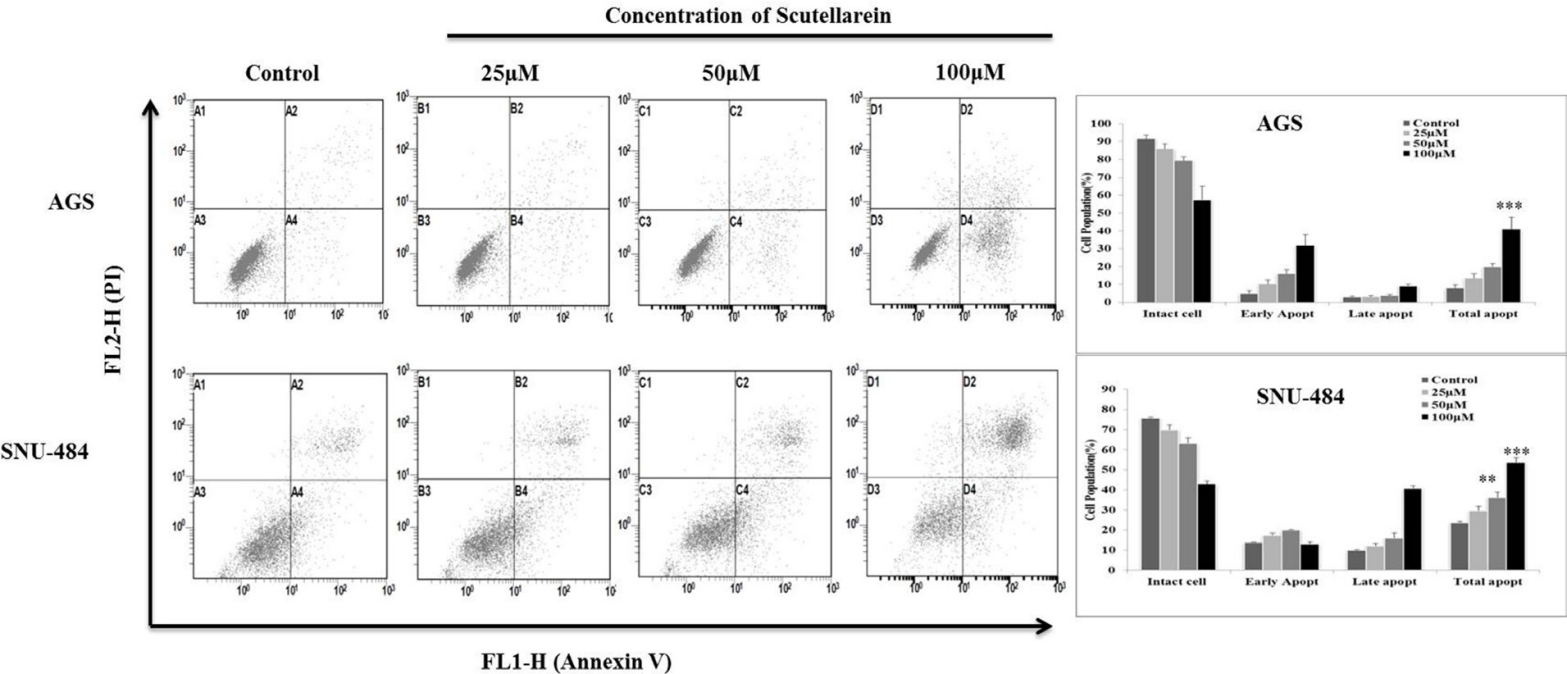
Scutellarein induces dose dependent apoptosis in AGS and SNU-484 cells AGS and SNU-484 cells were treated with indicated concentrations of scutellarein or 24 h. Apoptosis was assessed by Annexin V-PI double staining by using Cytomics FC 500 (Beckman Coulter, Brea, CA, USA) and the data were analyzed using CXP Software. The data are expressed as the mean ± standard deviation (SD) of at least three independent experiments. (^∗∗^*P* < 0.05 ^∗∗∗^*P* < 0.01 compared to control).

**Figure 4:**
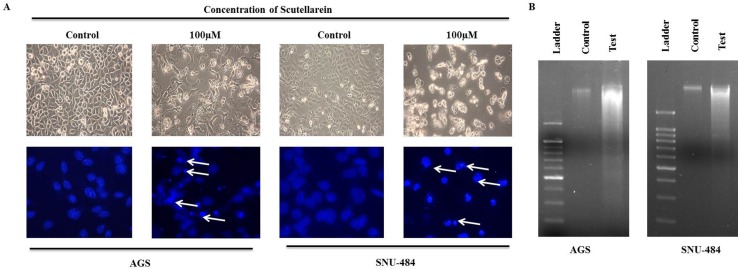
Morphology of cells treated with or without scutellarein (**A**) Morphology and DAPI Fluorescent staining of AGS and SNU-484 cells treated with or without scutellarein for 24h and was examined under light microscopy and fluorescence microscopy (×400). (White arrows showing bright blue regions indicate fragmented or condensed nuclei). (**B**) DNA ladder was examined in untreated and scutellarein treated AGS and SNU-484 cells. In scutellarein treated group we observed the DNA fragmentation compared to untreated group.

Since apoptotic cells with hypodiploid DNA content were detected in the sub-G1 phase of cell cycle, another apoptotic hallmark, phosphatidylserine exposure was evaluated by Annexin V-FITC/ PI staining using flow cytometry in both AGS and SNU-484 treated with the indicated concentration of Scutellarein for 24 h in order, to confirm whether Scutellarein induces apoptosis. Cytometric results revealed as showed in Figure 3, scutellarein induces dose-dependent apoptosis cell population in both the cells. After 24 h exposure to scutellarein, the increasing dose resulted in an increased proportion of total apoptotic cell by more than threefold, from 8% in the control to 41% in 100 μM Scutellarein; the early apoptotic population is being major in AGS cells. Whereas SNU-484 cells after 24 h treatment and in increasing dosag resulted in an increased proportion of total apoptotic cell by more than two fold, from 23% in the control to 54% in 100 μM scutellarein, the late apoptotic population is being major in SNU-484 cells. To confirm scutellarein induced cell death is apoptotic we conducted nuclear staining of both the cells with DAPI after 24 h scutellarein treatment. As shown in Figure 4A, bright blue regions indicate fragmented or condensed nuclei in treated group of cell in both AGS and SNU-484 cells indicating scutellarein causing apoptotic cell death. DNA fragmentation results revealed as showed in Figure 4B, a typical ladder pattern of fragmented DNA in the Scutellarein treated group compared to a control group with no pattern of fragmented DNA, which indicates internucleosomal cleavage associated with apoptosis. These results thus demonstrate the ability of scutellarein to induce cell death AGS and SNU-484 cells through apoptosis.

### Scutellarein induces mitochondrial apoptosis and regulate intrinsic pathway of apoptosis in gastric cancer cells

**Figure 5:**
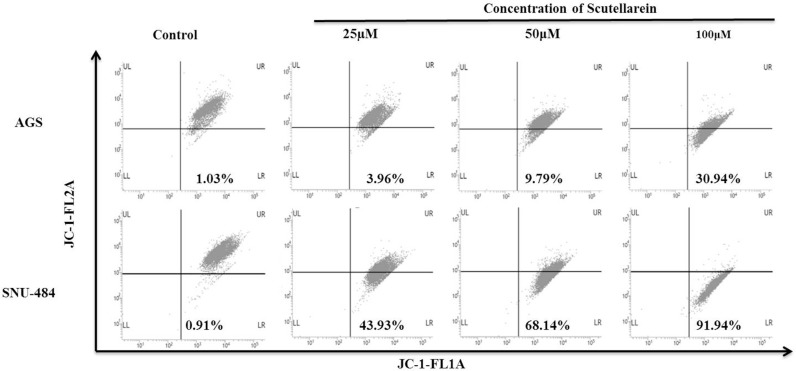
Flow cytometric analysis of the mitochondrial membrane potential (ΔΨm) AGS and SNU-484 cells were incubated with or without scutellarein at indicated concentrations of for 24 h at 37°C in complete media. After 24 h incubation cells were harvested and washed with 1X PBS. Washed cells were stained with JC-1 (5,5’,6,6’-tetrachloro-1,1’,3,3’-tetraethylbenzimidazolylcarbocyanine iodide) dye for 15 min (10 μg/ml) in dark and change in fluorescent intensity was analyzed using FACS Calibur flow cytometer (BD Biosciences, Franklin Lakes, NJ, USA).

**Figure 6:**
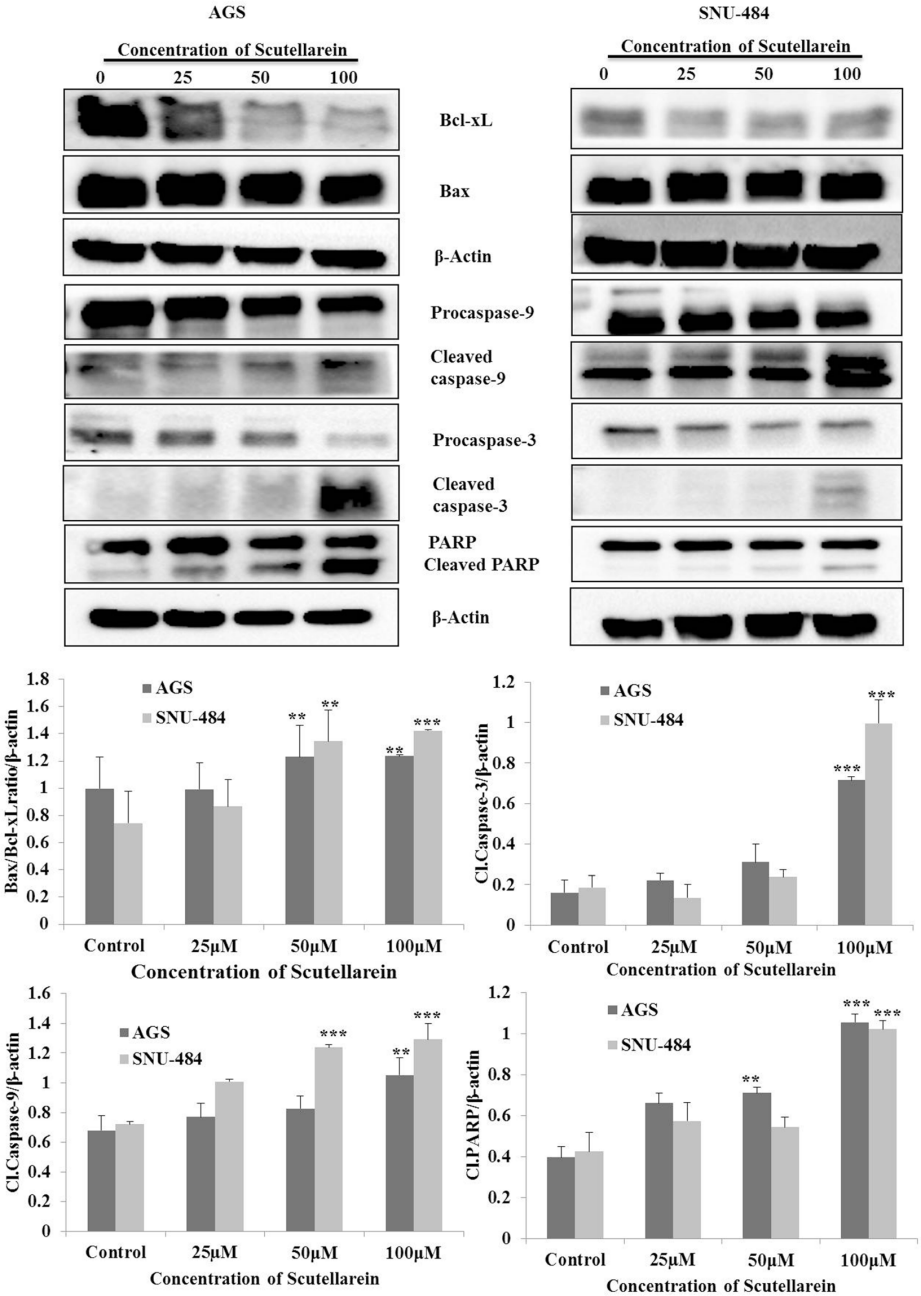
Caspases activation and subsequent cleavage of PARP in scutellarein -treated AGS and SNU-484cells AGS and SNU-484 cells were treated with indicated concentrations of scutellarein for 24 h. The cell lysates were subjected to SDS–PAGE and analyzed by immune-blotting. Densitometry analyses of Bax/Bcl-xL ratio, Cl.caspase-9, Cl.caspase-3 and Cl.PARP proteins expressions were expressed as the mean ± standard deviation (SD) of at least three independent experiments. (^∗∗^*P* < 0.05 ^∗∗∗^*P* < 0.01 compared to control).

Decline in the mitochondrial membrane potential (MMP, ΔΨm) is a characteristic of apoptosis. In the present study the mitochondrial ΔΨm was detected using fluorescence dye JC-1.The changes of fluorescence from red to green reflected the changes of mitochondrial ΔΨm from normal high ΔΨm to a low ΔΨm. As shown in Figure 5, Scutellarein remarkably increased the green fluorescence of JC-1 from 1.03% in control group to 30.94% (100 μM) in AGS cells and from 0.91% in control to 91.94% (100 μM) in SNU-484 cells, indicating ΔΨm reduction in a dose dependent manner. In addition as shown in Figure 6, the expressions of mitochondrial related apoptotic proteins, such as Bax and Bcl-2 were also analyzed in scutellarein treated AGS and SNU-484 cells. Scutellarein increased the expression of mitochondrial related protein Bax/Bcl-xL ratio of AGS and SNU-484 cells in a dose-dependent manner. To characterize the potential signaling pathway by which scutellarein induced apoptosis in AGS and SNU-484 cells, the modification in the expression levels of different apoptosis-regulating proteins such as initiator caspases (capase-9), effector caspases (capase-3), PARP (poly-ADP-ribose polymerase) were examined by western blotting. Both AGS and SNU-484 cells were treated indicated concentration of scutellarein for 24 h and protein samples were prepared and analyzed by western blotting. As shown in Figure 6, Pro-caspase-9 and -3 protein levels in both AGS and SNU-484 cells significantly declined in a dose-dependent manner and successive increase in the cleaved caspase-9 and -3. Scutellarein also induced an increase in the expression of cleaved PARP in both the cell lines at 24 h, which is significant compared to control group. Since the cleavage of PARP was observed, this confirms the activation of caspase-3 because PARP is a substrate of activated caspase-3. These results suggest that scutellarein induced apoptosis in both AGS and SNU-484 cells by up regulating Bax/Bcl-xL ratio via activation of the intrinsic pathway of caspases.

### Scutellarein decreases IAP proteins expressions and activates p53 by down regulating MDM2

**Figure 7:**
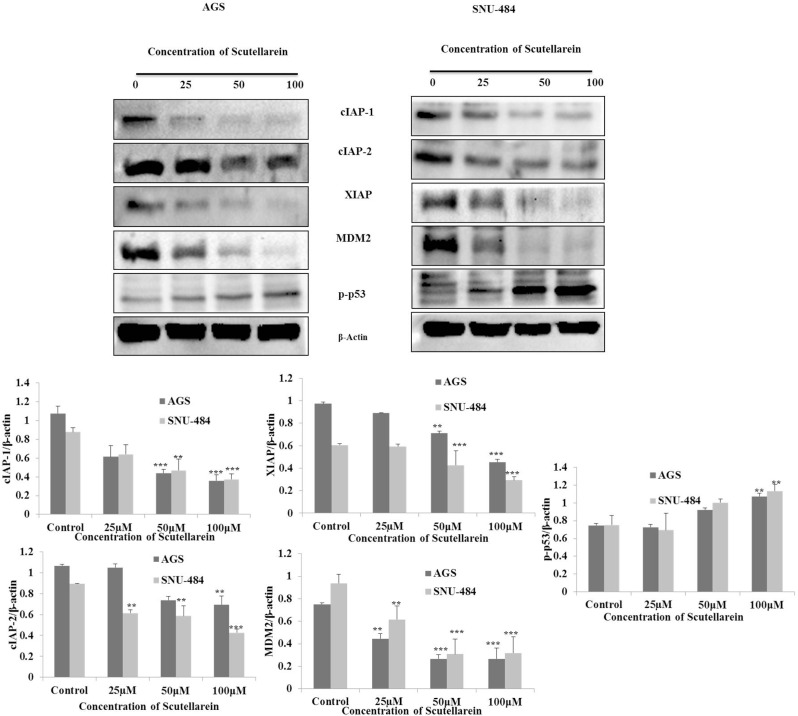
Down regulation of cIAP-1, cIAP-2, XIAP, MDM2 and activation of p53 AGS and SNU-484 were treated with indicated concentrations of scutellarein or 24 h. The cell lysates were subjected to SDS–PAGE and analyzed by immune-blotting. Densitometry analyses of cIAP-1,-2, XIAP, MDM2, p53 and p-p53 proteins expressions were expressed as mean ± SD of three independent experiments. (^**^P < 0.05 ^***^P < 0.01 compared to control).

Based on the result, it was originally hypothesized that scutellarein induces intrinsic pathway of apoptosis in both AGS and SNU-484 cells. Hence, we searched for the regulatory mechanism in which this intrinsic pathway of apoptotic proteins was activated after treated with scutellarein. We examined cIAP1, cIAP2, and XIAP expression by western blot which are apoptosis inhibitor proteins which mainly block caspases activation. Western blot was performed after treatment with 25, 50 and 100 μM scutellarein. As shown in Figure 7, three IAP protein expressions (cIAP1, cIAP2, and XIAP) were significantly decreased in a dose-dependent manner indicating scutellarein induced activation of caspases are due to decline in the expression of IAPs in both AGS and SNU-484 cells. The tumor suppressor p53 strong transcription factor that controls a major pathway that conserving cells from malignant transformation and tightly regulate the cancer cell apoptosis and interestingly the p53 expression are suppressed in most of the cancer cells. MDM2 is a negative regulator of p53, which blocks the activation of p53 in most of the cancer cells which leads to deregulation of apoptosis in cancer cells and it also increase the expression of XIAP protein expression. As shown in Figure 7, our western blot result revealed that a significant decline in the protein expression of MDM2 protein in both AGS and SNU-484 cells treated with scutellarein in a dose-dependent manner. Western blot result also revealed the consecutive activation of p53 as well as downregulation of XIAP in both the cell lines treated with Scutellarein. Collectively our result indicated that apoptosis induced by scutellarein in both AGS and SNU-484 cells are regulated by p53 protein.

### Scutellarein induced caspase-dependent apoptosis in AGS Cells and p53 independent apoptosis in SNU-484 Cells

**Figure 8:**
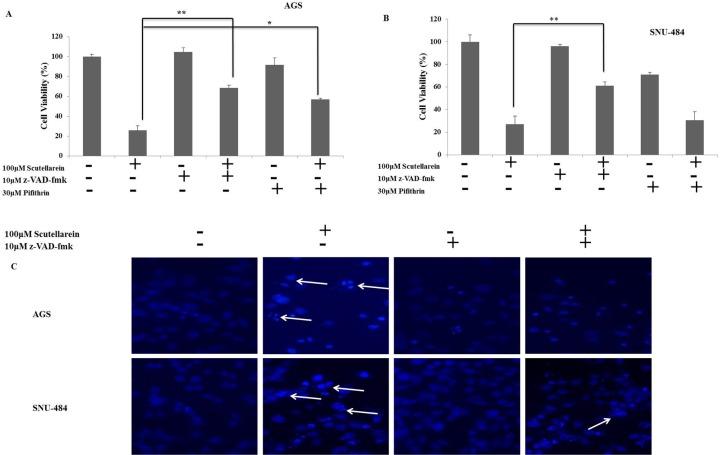
Inhibitor assays (**A, B**) Cell viability of AGS and SNU-484 cells with or without z-VAD (z-VAD- fmk) or Pifithrin-α.AGS and SNU-484 cells were treated with 10 μM of z-VAD- fmk and 30μM of Pifithrin for 1 h before scutellarein treatment. The cell viability was determined by MTT assay. (**C**) DAPI Fluorescent staining of AGS and SNU-484 cells treated with or without 20 μM of z-VAD- fmk and co-treated with scutellarein for 24h and was examined under light microscopy and fluorescence microscopy (×400).

**Figure 9:**
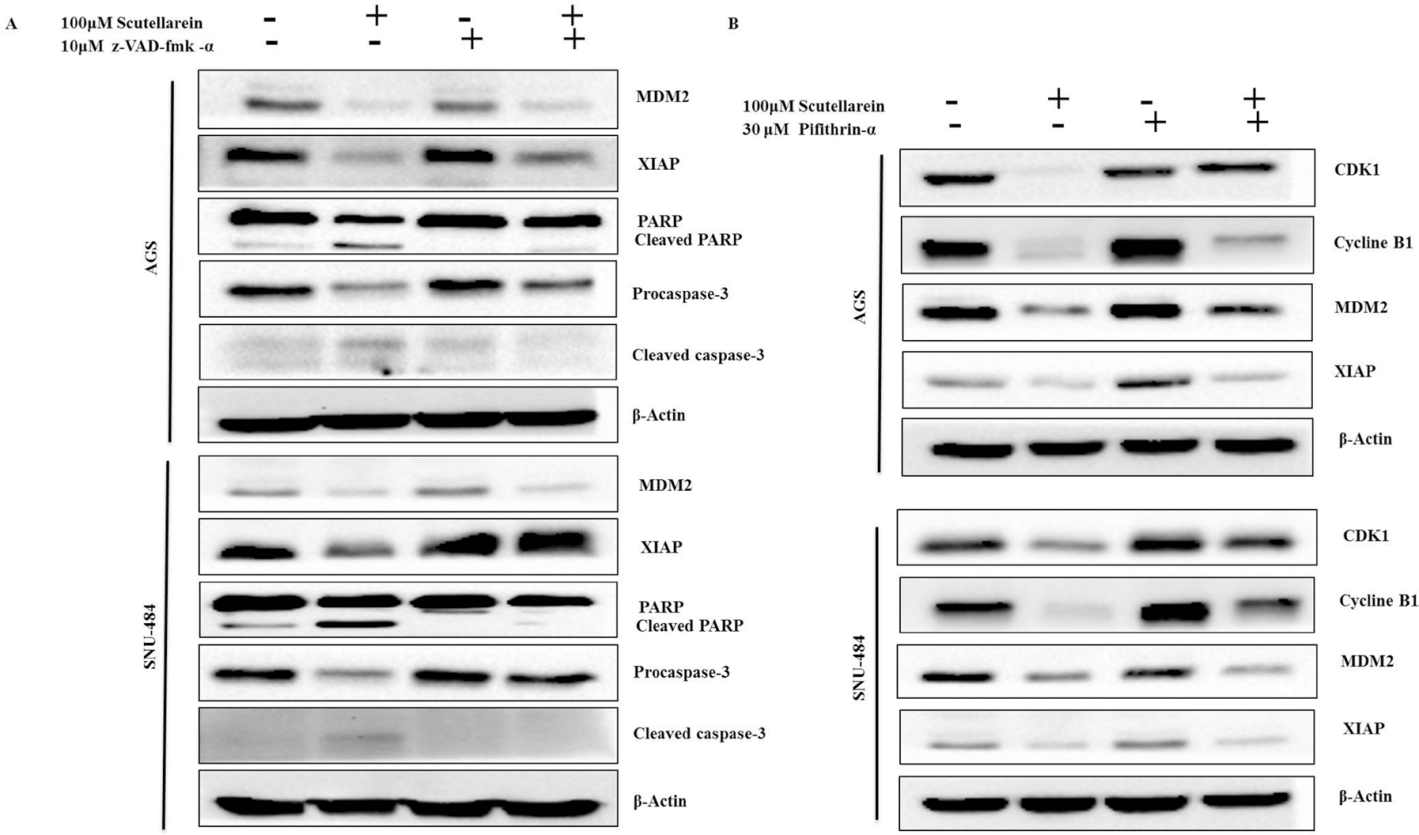
Effect of caspase inhibitor and p53 inhibitor on scutellarein-induced apoptosis in AGS and SNU-484 cells (**A**) The cells were incubated with broad-spectrum caspase inhibitor z-VAD-fmk at 10 μM concentration or (**B**) Pifithrin-α as a p53 inhibitor at 30 μM concentration 1 h prior to Scutellarein treatment. After the incubation with 100 μM Scutellarein for 24 h, whole cell lysates were subjected to immunoblotting.

To further confirm the role of caspases and p53 in scutellarein induced apoptosis in AGS and SNU-484 cells we conducted an inhibitory assay with z-VAD-fmk (caspase inhibitor) and pifithrin-α (p53 inhibitor). Both AGS and SNU-484 cells were pretreated with or without z-VAD-fmk/ pifithrin-α for 1 h followed by 100 μM of Scutellarein for 24 h and cell viability was measured by MTT assay. As shown in Figure 8A and B, scutellarein induced cell cytotoxicity in both AGS and SNU-484 gastric cancer cells were partially recovered by pretreatment with pan-caspase inhibitor z-VAD-fmk as compared with scutellarein treated cells, confirming caspase-dependent apoptosis in both AGS and SNU-484 cells. In contrast, Scutellarein induced cytotoxicity also recovered in the pifithrin-α pretreated AGS cells which are significant compared to the scutellarein treated group, whereas in the case of SNU-484 cells the cytotoxicity caused by scutellarein has been increased in pifithrin-α pretreated and cotreated cells. These results confirmed by DAPI stain and western blot of AGS and SNU-484 cells pretreated with pan-caspase inhibitor z-VAD-fmk and co-treated with Scutellarein. DAPI results revealed that nuclear condensation in scutellarein treated cells with pretreated pan-caspase inhibitor z-VAD-fmk are drastically reduced compared to scutellarein alone treated a group of both AGS and SNU-484 cells, revealing scutellarein induced apoptosis is reversed by blocking caspase activity as shown in Figure 8C. Western blot results revealed as shown in Figure 9, MDM2 and XIAP protein expression is regained in AGS cells pretreated with pan-caspase inhibitor z-VAD-fmk compared to scutellarein alone treated group. In SNU-484 cell, MDM2 protein expression was reduced similar to the scutellarein treated group. Whereas, XIAP expressions are regained in pretreated pan-caspase inhibitor z-VAD-fmk compared to scutellarein alone treated group. Cleaved PARP and cleaved caspase-3 protein expressions are significantly reduced in scutellarein treated cells with pretreated pan-caspase inhibitor z-VAD-fmk compared to scutellarein alone treated group (Figure 9A). Simultaneously, the procaspase-3 expression is increased in scutellarein treated cells with pretreated with pan-caspase inhibitor z-VAD-fmk compared to scutellarein alone treated group. As shown in Figure 9B, western blot results of AGS and SNU-484 cells pretreated with Pifithrin-α a p53 inhibitor revealed, CDK1, Cyclin B1and MDM2 protein expressions are regained and XIAP protein expressions are decreased compared to scutellarein alone treated group in AGS cells. Whereas CDK1 and Cyclin B1 protein expressions are regained and MDM2 and XIAP protein expressions are decreased compared to scutellarein alone treated group in SNU-484 cells. Our results confirm that scutellarein induced apoptosis in AGS and SNU-484 cells are caspase-dependent and independent of p53 specifically in SNU-484 cells.

## DISCUSSION

Several natural flavonoids compounds derived from plants were recently reported to cause cancer cell death by induction of apoptosis in various human cancer cells [22, 28, 29]. The aims of this study were to determine the anti-cancer effects of scutellarein against human gastric cancer cells and its potential mechanisms of action. The present report thus illustrates the underlying mechanisms by which scutellarein induces apoptosis in the gastric cancer cells. Scutellarein inhibited the proliferation of AGS and SNU-484 cells in both doses- and time-dependent manner. In addition,it caused cell-type specific G2/M phase growth arrest in SNU-484 cells, whereas it causes an increase in Sub-G1 population in AGS cells treated with scutellarein indicating apoptosis. Furthermore, scutellarein increased the number of total apoptotic cells as demonstrated by Annexin-V-FITC/PI assay. It inhibited the expression of anti-apoptotic Bcl-2 family members such as Bcl-xL and activated pro-apoptotic proteins like Bax. It further activated caspase-9 which subsequently induced caspase-3 and, resulting in PARP cleavage. It was also identified that the inhibition of caspases by the general caspase inhibitor, z-VAD-fmk, blocked Scutellarein -induced apoptosis in both AGS and SNU-484 cells, but the SNU-484 cells were not spared from scutellarein-induced apoptosis when p53 transcriptional activity was suppressed by pifithrin-α, perhaps Scutellarein induced cytotoxicity was recovered in the pifithrin-α pretreated AGS cells.

Apoptosis, a highly controlled mode of cell death, is utilized to eliminate superfluous, aged, injured or infected cells from the body [30]. Mitochondria play a vital role in apoptosis by regulating apoptotic (Bax, Bak) and antiapoptotic protein (Bcl-2,Bcl-xL) ratio which leads to the activation of caspases [31]. Caspases, a family of aspartic acid-specific proteases, are the major effectors of apoptosis. To diminish their activity, caspases are normally synthesized as inactive precursors but become activated at the onset of apoptosis by activation signals. Once active, caspases supervise over the ordered eliminating of the cell through restricted proteolysis of hundreds of substrate proteins [12]. Caspases also affect the cytoskeletal structure, cell cycle regulation, and signaling pathways, ultimately leading to the morphologic manifestations of apoptosis, such as DNA condensation and fragmentation, and membrane blebbing. The current study results demonstrated that increased ratio of Bax/Bcl-xL in both AGS and SNU-484 cells treated with scutellarein in a dose-dependent manner and subsequent activation of caspase-9 and caspase-3 which leads to cleavage of PARP leads to primary substrate of cleaved caspase-3.

The apoptotic pathways are regulated by proteins such as the tumor suppressor p53 and apoptotic inhibitor proteins (IAPs) which are tightly regulated by MDM2 protein (Mouse double minute 2 homolog) through an autoregulatory feedback loop [6, 9, 32]. MDM2 binds to p53 tumor suppressor protein with high affinity and negatively modulates its transcriptional activity and stability [33]. Abundant amount of studies have shown that overexpression of MDM2 found in sevral human tumors cells, which effectively impairs p53 function. Inhibition of MDM2 expression stabilize p53 and leads the cancer cells to cancer therapy [34]. Increased expression of IAPs leads to resistance to chemotherapeutic apoptosis in cancer cells by blocking caspase activation [35]. Study’s also demonstated that interaction of XIAP and MDM2 inhibit the ability of MDM2 as self-association and self-ubiquitination, which upregulate the MDM2 protein stabilization and cancer cell surivival [36]. Many recent reports have demonstrated that targeting p53-MDM2 interaction and inhibiting IAP family of proteins in cancer cells to overcome resistance caused to cancer chemotherapy apoptosis [37]. Contrary to these reports, our present study clearly demonstrated that down-regulation of IAP family proteins (cIAP1, cIAP2, and XIAP) leads to activation of caspase-9 and caspase-3 in both AGS and SNU-484 cells in a dose-dependent manner significant compared to untreated group and scutellarein treated group of cells. Caspase inhibitor assay results revealed that the inhibition of caspases by the general caspase inhibitor, z-VAD-fmk, blocked Scutellarein -induced apoptosis in both AGS and SNU-484 cells confirming Scutellarein induced apoptosis in gastric cancer cells are caspase-dependent. As a tumor suppressor gene, p53 functions as a transcription factor and stimulates the expression of many apoptotic effectors, such as PUMA, NOXA, BID, Bax, p53AIP1 proteins. The p53 can also transcriptionally repress the expression of anti-apoptotic proteins, such as Bcl-2, Bcl-XL, and surviving [8, 38]. In our study, scutellarein treatment remarkably decreased the expression of MDM2 which leads increased the p53 expression levels in a time-dependent pattern in AGS and SNU-484 cells. As expected, the increased p53 protein level was correlated with the up regulation of its downstream target gene Bax and the down regulation of Bcl-xL. Such an effect likely forms the basis for the induction of apoptosis of AGS and SNU-484 cells. These findings are consistent with the previous results reported [39, 40]. Our inhibitory assay results show that, Scutellarein induced cytotoxicity recovered in the Pifithrin-α pretreated AGS cells which are significant compared to scutellarein treated group, whereas in the case of SNU-484 cells the cytotoxicity caused by Scutellarein has been increased in Pifithrin-α pretreated and co-treated cells indicating scutellarein induced p53 dependent apoptosis in AGS cells whereas in SNU484 cells are independent of p53 activated apoptosis. These results are supported by western blot results, whereas, XIAP protein expression are regained in regained in pretreated pan-caspase inhibitor z-VAD-fmk compared to scutellarein alone treated group suggesting the involvement of XIAP in caspase dependent cell death in Scutellarein treated AGS and SNU-484 cells. Western blot results of p53 inhibitor assay unveil that, CDK1, Cyclin B1and MDM2 protein expressions are regained and XIAP protein expressions are decreased compared to scutellarein alone treated group in AGS cells. Whereas, CDK1 and Cyclin B1 protein expressions are regained and MDM2 and XIAP protein expressions are decreased compared to scutellarein alone treated group in SNU-484 cells.

**Figure 10:**
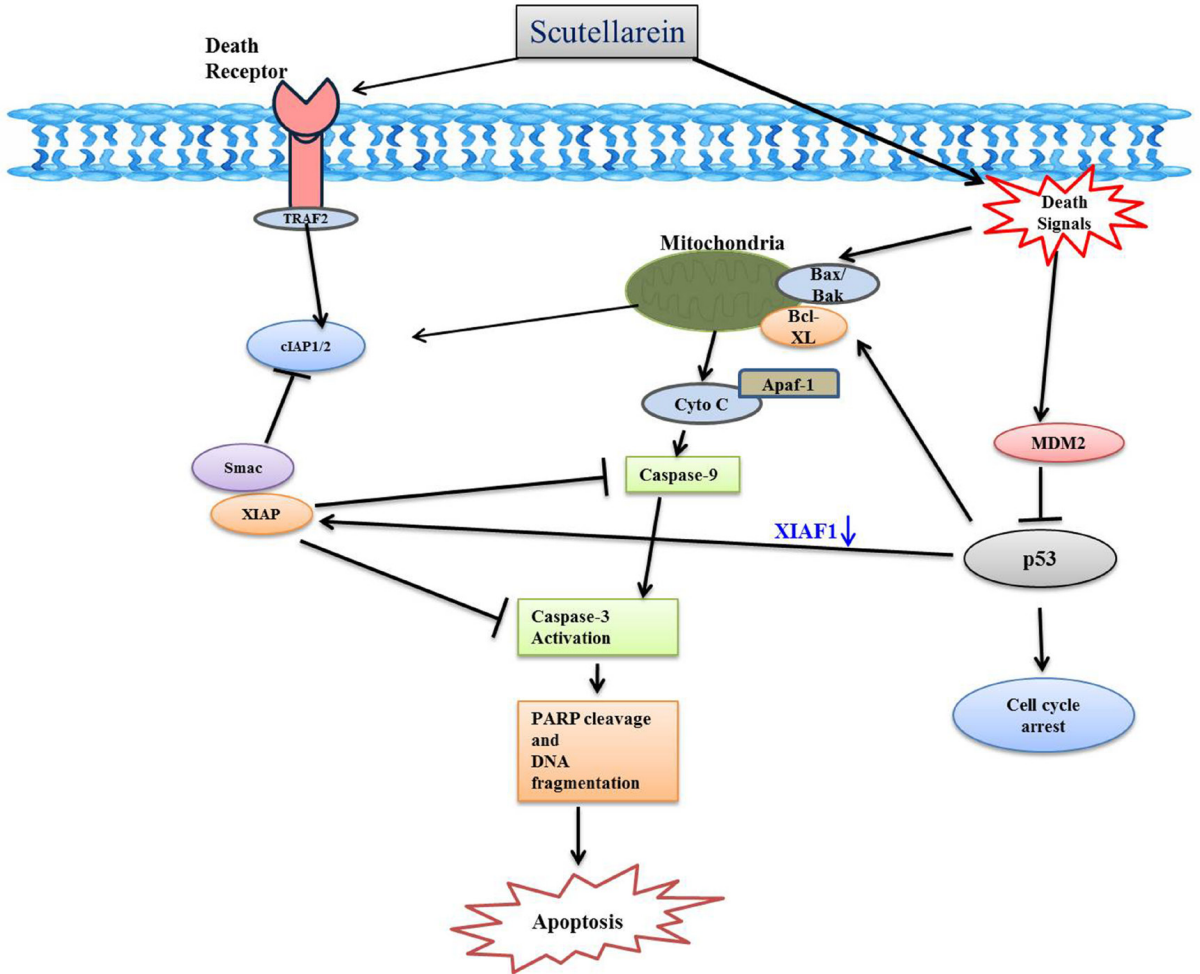
Schematic diagram showing the mechanisms underlying the anti-cancer effects of scutellarein in AGS and SNU-484 cells gastric cancer cells

In summary, this study demonstrated that the scutellarein on AGS and SNU-484 cells significantly inhibits cell proliferation and induces apoptotic cell death via down regulated MDM2 which activated the tumor suppressor protein p53 and downregulate the IAP family proteins (cIAP1, cIAP2, and XIAP), leads to caspase-dependent apoptosis in AGS and SNU-484 cells (Figure 10). Our data clearly demonstrate that Scutellarein can be further evaluated as a potential anticancer agent for gastric cancer.

## MATERIALS AND METHODS

### Chemicals and reagents

AGS and SNU-484 human gastric cancer cells obtained from the Korea Cell Line Bank (Seoul, Korea) were cultured in RPMI 1640 medium supplemented with 10% (v/v) fetal bovine serum (FBS) from GIBCO (BRL Life Technologies, Grand Island, NY, USA), 100 U/mL penicillin, and 100 μg/mL streptomycin at 37 °C in a humidified atmosphere of 95% air and 5% CO_2_. Scutellarein compound purchased from Chengdu Biopurify Phytochemicals Ltd (Chengdu, Sichuan, China, 611130). 3-(4,5-Dimethylthiazol-2-yl)-2,5-diphenyltetrazolium bromide (MTT) was obtained from Sigma–Aldrich (St. Louis, MO, USA). Materials and chemicals used for electrophoresis were obtained from Bio-Rad (Hercules, CA, USA). Primary antibodies to Bcl-xL, Bax, Caspase (3,-9), cleaved caspase (3,-9), poly ADP-ribose polymerase (PARP), cleaved-PARP, p53, p-p53, cdc25c, Cyclin B1, CDK1 and β-actin were purchased from Cell Signaling Technology (Danvers, MA, USA). Primary antibodies MDM2, XIAP, cIAP-1 and cIAP-2 were purchased from Santa Cruz Biotechnology (Dallas, Texas 75220 U.S.A.) Horseradish peroxidase-(HRP-) coupled goat anti-mouse IgG and anti-rabbit IgG were purchased from Enzo Life Sciences. Pifithrin-α and Propidium iodide (PI) were purchased from Sigma–Aldrich (St. Louis, MO, USA). DAPI (4′, 6-Diamidino-2-phenylindole) purchased from Vector Laboratories Inc. (Burlingame, CA, USA). z-VAD-fmk was purchased from Enzo Life Sciences, Inc. (Farmingdale, NY, USA). 6× Agarose Gel Loading Buffer was purchased from Bioneer (Daejeon, Korea), DNA marker was purchased from iNtRON Biotechnology (Kyungki-Do, Korea). JC-1 dye was purchased from Molecular Probes, Inc. (Eugene, QR, USA).

### Cell viability assay

Cell viability assay was performed using MTT assay, for which, AGS and SNU-484 cells were seeded at a density of 1 × 10^5^ cells/well in 12 well plates and then treated with 25, 50, 75 and 100 μM of scutellarein or vehicle (DMSO) alone for 24 and 48 h. After incubation, 100 mL of 0.5% (*w*/*v*) MTT dissolved in 1× PBS was added to each well and incubated for 3 h at 37 °C in the dark. The formazan contained in the cell was solubilized by 500 μL of DMSO and the absorbance was measured at 540 nm with an enzyme-linked immunosorbent assay (ELISA) plate reader (BioTek Instruments Co., Seoul, Korea).

### Flow cytometry analysis for cell cycle analysis

AGS and SNU-484 cells were collected after incubation with scutellarein at the indicated concentrations (0, 25, 50, and 100 μM) for 24 h. After incubation, cells were washed with ice-cold PBS, trypsinized and collected in a 15 mL conical tube and pelleted by centrifugation (1000× *g*) for 5 min. The cells were then fixed in 70% (*v*/*v*) ethanol for 1 h at 4°C. The cells were washed with PBS and stained with Propidium iodide (50 μg/mL) including RNaseA (0.1 mg/mL) in PBS (pH 7.4) for 30 min in the dark. Flow cytometry analyses were performed with Cytomics FC 500 (Beckman Coulter, Brea, CA, USA). In each sample, 10,000 cells were sorted. The data were analyzed by using CXP Software (Beckman Coulter, Inc., Fullerton, CA, USA)

### Annexin V-propidium iodide apoptosis detection

Apoptotic cells were detected by using an FITC Annexin-V apoptosis detection kit 1 (BD Pharmingen, San Diego, CA, USA). Briefly, after treatment with various concentrations of scutellarein (0, 25, 50 and 100 μM), for 24 h, the cells were collected and washed with PBS, re-suspended in binding buffer, and stained with Annexin V-FITC and PI for 15 min at room temperature in the dark, prior to the addition of binding buffer. Flow cytometry analyses were performed with Cytomics FC 500 (Beckman Coulter, Brea, CA, USA). In each sample, 10,000 cells were sorted. The data were analyzed by using CXP Software (Beckman Coulter, Inc., Fullerton, CA, USA).

### Nuclear staining with DAPI

After 24 h incubation with Scutellarein at the indicated concentrations, cells were washed with phosphate-buffered saline (PBS). Cells were fixed with 37% formaldehyde and 95% ethanol (1:4) for 10 min at room temperature followed by washing the cells with PBS and stained with 2.5 μg/mL of DAPI solution for 10 min at room temperature (RT). The stained cells were then examined through a fluorescence microscope from Leica Microsystems Ltd. (Seoul, Korea).

### Dna fragmentation assay

Both AGS and SNU-484 after 24 h incubation with 100 μM Scutellarein, the cells were harvested and lysed in a lysis buffer (1% NP-40 in 20 mM EDTA, 50 mM Tris-HCl, and pH 7.5) for 30 s at RT (room temperature). Centrifuged the lysed cells at 3000 rpm for 5 min and supernatant was collected. Pooled supernatant was incubated for 2 h at 56 °C after adding 10 μL of 10% SDS solution (final concentration: 1% SDS) and 10 μL of 50 mg/mL RNaseA (final concentration 5 μg/μL). Then, 10 μL of Proteinase K (final concentration 2.5 μg/μL) was added and incubated for 2 h at 37 °C, followed by adding 1/2 volume (65 μL) of 10 M NaCl and 2.5 volume (500 μL) of ice-cold ethanol and mixed thoroughly. The mixture was incubated for 1h at −80°C (ethanol precipitation). It was then centrifuged for 20 min at 12,000 rpm followed by washing the white pellet with 200 μL of 80% ice-cold ethanol and air-dried for 10 min at room temperature. The pellet was dissolved in 50 μL of TE buffer and the concentration of DNA was determined using Nanodrop. Electrophoretic analysis was performed on 1.5% agarose gels containing 0.1μg/mL EtBr (ethidium bromide) .Tris acetate EDTA was used as the electrophoresis running buffer and DNA bands were visualized by UV light and documented by photography.

### Analysis of mitochondrial membrane potential (MMP, ΔΨm)

JC-1 (Molecular Probes, Inc. Eugene, QR, USA) was dissolved in DMSO (5 mg/mL). AGS and SNU-484 cells were incubated with or without scutellarein at indicated concentrations of for 24 h at 37°C in complete media. After 24 h incubation cells were harvested and washed with 1X PBS. Washed cells were stained with JC-1 (5,5’,6,6’-tetrachloro-1,1’,3,3’-tetraethylbenzimidazolylcarbocyanine iodide) dye for 15 min (10 μg/ml) in dark and change in fluorescent intensity was analyzed using FACS Calibur flow cytometer (BD Biosciences, Franklin Lakes, NJ, USA).

### Inhibitor assay

To confirm the role of p53 and caspases in scutellarein induced apoptosis, inhibitor studies were carried out. The cells were incubated with Pifithrin-α as a p53 inhibitor at 30μM concentration or broad-spectrum caspase inhibitor z-VAD-fmk at 20 μM concentration 1 h prior to Scutellarein treatment. After the incubation with 100 μM scutellarein for 24 h, the cells were processed for cell viability with MTT assay, DAPI staining was used to measure the apoptosis and western blot analysis to confirm the apoptotic related proteins from cells pretreated with Inhibitor of caspase and p53 and co-treated with Scutellarein.

### Western blot analysis

AGS and SNU-484 cells were treated with Scutellarein at 0, 25, 50 and 100 μM for 24 h at 37 °C and were lysed in RIPA buffer [1% (w/v) NP40, 1% (w/v) sodium deoxycholate, 0.1% (w/v) SDS, 0z.15 M NaCl, 0.01 M sodium phosphate buffer, pH 7.2, 2 mM EDTA, and 50 mM phosphatase inhibitor cocktail]. The cell extracts were centrifuged for 30 min at 12,000 rpm to remove debris. Bradford assay (Bio-Rad) method was used to quantify proteins. Proteins were separated by 8%–12% SDS-polyacrylamide gel electrophoresis (SDS-PAGE) and transferred to a polyvinylidene fluoride (PVDF) membrane (Immunobilon-P, 0.45 mm; Millipore, Billerica, MA, USA) using the TE 77 Semi-Dry Transfer Unit (GE Healthcare Life Sciences, Buckinghamshire, UK). The membranes were blocked with 5% non-fat skim milk in Tris-buffered saline containing 1% Tween 20 (TBS-T, pH 7.4) at room temperature for 1 h, and incubated overnight at 4°C with a range of 1:200 to 1:1000 dilution of respected primary antibody. The membranes have washed five times with TBS-T for 10 min each at room temperature, incubated with a 1:2000 dilution of HRP conjugated secondary antibody for 3 h at room temperature. The membranes were then rewashed five times with TBS-T. Blots were developed with ECL detection system (GE Healthcare Life Science). The bands were quantitatively analyzed by using the Image J program (http://rsb.info.nih.gov). The densitometry readings of the bands were normalized according to β-actin expression.

### Statistical analysis

Each experiment was performed in triplicate and data were expressed as the mean ± standard deviation (SD). Differences between the control and Scutellarein treated groups were determined using one-way analysis of variance followed by a student’s *t*-test with *p* < 0.05 and *p* < 0.01 as the limit of significance. All statistical analyses were performed using SPSS software (SPSS for Windows, v. 10.0; SPSS Inc., Chicago, IL, USA).
